# Amelioration of Diabetic Nephropathy by Targeting Autophagy via Rapamycin or Fasting: Relation to Cell Apoptosis/Survival

**DOI:** 10.3390/cimb43030120

**Published:** 2021-10-22

**Authors:** Khaled Gouda, Sherihan AbdelHamid, Ahmed Mansour, Nesreen Omar, Hala El-Mesallamy

**Affiliations:** 1 Biochemistry Department, Faculty of Pharmacy, Modern University for Technology and Information, Cairo 12055, Egypt; K_Goudaph@hotmail.com (K.G.); dr.n.nabil@gmail.com (N.O.); 2 Biochemistry Department, Faculty of Pharmacy, Ain-Shams University, Cairo 11566, Egypt; dr.sherehan@pharma.asu.edu.eg; 3 Pharmacology and Toxicology Department, Faculty of Pharmacy, Al-Azhar University, Cairo 11651, Egypt; dr.ahmedmmansour@yahoo.com; 4 Dean of Faculty of Pharmacy, Sinai University, North Sinai 45518, Egypt

**Keywords:** autophagy, β-cell, P-glycoprotein, apoptosis, DM, STZ, fasting, rapamycin, mTOR

## Abstract

Autophagy has been demonstrated to have a beneficial effect on diabetic nephropathy (DN). Rapamycin, an inhibitor of mTOR, was shown to stimulate β-cell autophagy. However, its effects on preventing or ameliorating DN is unclear, and its effects are worth studying. As fasting is now an attractive protective strategy, we aim to compare its effect to rapamycin effects on pancreatic and renal cells. Twenty-eight adult male Wistar Albino rats were randomly divided into four groups, using streptozotocin (STZ) to induce diabetes mellitus (DM). Autophagy was induced by two ways; rapamycin or fasting. The extent of autophagy and apoptosis were investigated by measuring the level of LC3B and p53 proteins, respectively, in pancreatic and kidney tissues using Western blotting (WB) technique and imaging the renal cells under transmission electron microscope. The efflux transporter P-glycoprotein was quantified by WB as well. Rapamycin-induced autophagy occurred concurrently with apoptosis. On the other hand, fasting supported P-glycoprotein recovery and renal cell survival together with disabling β-cells apoptosis. In conclusion, this study provides a potential link between rapamycin or fasting for the cross-regulation of apoptosis and autophagy in the setting of cell stress as DN. Unlike rapamycin, fasting enhanced the active expression of ABCB1 efflux protein, providing insights on the potential ameliorative effects of fasting in DN that require further elucidation.

## 1. Introduction

β-cells of islets of Langerhans function, structure, and mass maintenance are autophagy-based [1,2]. In addition, dysregulation of mitophagy affects insulin sensitivity, as mitochondrial dysfunction has been implicated in insulin resistance [3]. Autophagy is a self-degradative process achieved by lysosomes for removing aggregated proteins and damaged organelles [4].

It has been observed recently that calorie restriction by means of intermittent fasting and periodic fasting prolongs lifespan and reduces incidence of many age-associated diseases—such as cardiovascular diseases, cancer, kidney diseases and diabetes mellitus (DM) [5]. Compelling evidence has shown the role of fasting in adaptive cellular responses that protect against inflammation and oxidative damage and optimize energy metabolism [6]. It was shown that fasting mediates the activity of the mechanistic target of rapamycin (mTOR), resulting in induction of autophagy and cell repair mechanisms, while mitochondrial biogenesis and life span are increased experimentally [7,8,9]. Although autophagy may be apparently thought of as a survival mechanism, it has been shown to play a pivotal role in diabetic complications and hence could be considered as a potential therapeutic target for amelioration of these complications [10]. 

Upregulation of autophagy induced β-cell dysfunction with associated downregulation of insulin production and apoptosis of β-cells [11]. Consequently, it has been stated that the use of an autophagy inhibitor abrogated these effects and may promote islet function and survival [11].

The most well-known stimulator of autophagy is the drug rapamycin [12]. mTOR—a serine/threonine protein kinase—regulates important cellular processes, including growth, protein synthesis, and transcription. Being the central signaling molecule in determining autophagy levels in cells, mTOR kinase activation leads to inhibition of autophagy [13]. 

Rapamycin, a macrolide fungicide with immunosuppressant and antiproliferative properties, achieves its effects via binding to its intracellular receptor FK506-binding protein 12 (FKBP-12). The formed complex FKBP-12-rapamycin-associated protein 1 (FRAP1) binds to mTORC1 which possesses higher affinity toward this complex than mTORC2 [14] and interferes with the phosphorylation of ribosomal protein S6 kinase (S6K) at Thr389 [15]. The outcome of this repression effect is the disruption of critical processes regulated by the mTORC1 such as glucose metabolism, ribosome biogenesis, and autophagy [15]. As deregulation of the mTOR pathway has been implicated in DM [16], rapamycin, a specific inhibitor of mTOR, would be useful in preventing or ameliorating DM-complications. 

One important enzyme for autophagic process is autophagy related 3 (Atg3), an E2 enzyme for the microtubule-associated protein light chain 3 (LC3) lipidation process. Thereby, anchoring this enzyme to the membrane is essential for autophagocytosis [17].

Apoptosis is a mechanism by which cells that are superfluous, inefficient, ectopic, aged, weakened, unattached, or mutated are removed. The expression of p53 is related to both autophagy and apoptosis regulation [18]. The mechanism by which pancreatic and renal cellular processes are either activated or limited remains to be both controversial and to be explored. Moreover, autophagy is regulated by an intricate signaling network of cascades that have not yet been completely disentangled.

Accumulating evidence indicated that p53 can modulate autophagy in dual fashion, depending on its subcellular position. At the one side, p53 serves as a catalyst for nuclear transcription and transactivates pro-apoptotic, cell cycle-arresting, and pro-autophagic genes. Cytoplasmic p53, on the other hand, can function at mitochondria to facilitate cell death and can repress autophagy via poorly characterized mechanisms [19].

Because mTOR dysregulation occurs in DM, there are ongoing biochemistry efforts to target mTOR signaling for protecting against diabetic-associated complication(s) [20,21] Systemic administration of rapamycin ameliorates diabetes-induced renal dysfunction, a hypothesis to be examined in the current study.

Oxidative stress/antioxidant dysfunction, proteomic/hormonal alterations, and metabolic distresses promote diabetic nephropathy (DN) development [22]. DN is a severe complication of DM and the most noteworthy causative factor of end-stage renal failure [23]. Renal expression of ATP-binding cassette sub-family B member 1 (ABCB1), a cell membrane-bound glycoprotein that pumps foreign substances out of cells, could enhance the protective mechanism in the glomerular filtrate [24].

Cell survival could be enabled by autophagy, a cellular restoration process attempts to deliver damaged cellular cargo to lysosomes for degradation [25]. Under stressful conditions, the lysosomal system undergoes its protective response, in part, by redistribution of some membrane-bound proteins from the cell surface to intracellular organelles [26]. Among these membrane proteins, ABCB1 is the best-characterized efflux transport protein that plays vital cellular functions, including pancreatic β-cells and proximal tubular renal cells [27]. Knowing that, among the positive regulators of ABCB1, is the insulin hormone [28]; therefore, in type 1DM, where oxidative stress is predominant and insulin is lacking, the expression of ABCB1 is questionable. As autophagy is defective in diabetic kidneys [21], the extent of recycling ABCB1 through experimentally stimulating this defective diabetic-autophagic-setting is one of the study aims. 

One of the factors that negatively regulates autophagy in diabetic kidneys is mTOR [29] that is activated by excessive levels of nutrients, such as glucose, to impair autophagic activity [30]. Accordingly, this study is established to uncover the mystery regarding autophagy role in preventing DN; one of DM-related complications, provided the assumption, of autophagy beneficial effect may be gained, if the correct stimulus is used, where one of the examined stimuli is fasting [31]. Besides, the aim of this study is to assess the reparative effect(s) of rapamycin and fasting on pancreatic cells in STZ-treated-rats and also, follow the sequel on renal tissue.

## 2. Materials and Methods

### 2.1. Ethics Statement

Animals were housed in accordance with the principles of laboratory animal care NIH publication no. 85–23, http://grants1.nih.gov/grants/olaw/references/phspol.htm, revised 1985 (accessed on 20 December 2017). The experimental protocol was approved by the Ethical Committee of the Faculty of Pharmacy, Ain Shams University (37, July 2016).

### 2.2. Drugs and Chemicals

Rapamycin was purchased from GuangZhou Medcan Pharmatech Ltd. (GuangZhou, China); STZ from Sigma-Aldrich Chemical Com (St. Louis, MO, USA); Other biochemical reagents, unless otherwise specified, were purchased from Sigma-Aldrich Chemical Co. (St. Louis, MO., USA) and were of analytical grade.

### 2.3. Animals

The whole study was performed on 28 adult male Albino rats of Wistar strain weighing (150–200 g), obtained from the breeding colony of Helwan farm belonging to the Holding company for Biological products and Vaccines (Vacsera, Giza, Egypt).

### 2.4. Experimental Design

28 animals were randomly divided into 4 groups (7 rats/group); control group (C), STZ positive control (STZ), rapamycin-treated STZ (R-STZ), and fasting-STZ (F-STZ). All animal groups, except the fasting group, had ad libitum access to 30 g chow daily for maintaining the healthy state of their body tissues [32]. Rapamycin, was injected for three days in a dose 1 mg/kg body weight [33], prior to exposure to STZ for the R-STZ group. A fasting strategy was applied to induce autophagy [34]; rats of the fasting group were initially fed with 30 g chow on day 1 of the experiment then were subjected to gradual decrease (−2 g/2 days) in the amount of chow fed to them to be 15 g/day on the 15th day. On the 16th day, fasting rats were subjected for two weeks response to a day after day complete deprivation of food and water was ad libitum [34,35,36].

### 2.5. Preliminary Pilot Studies for Rapamycin Dosage Optimization

Rapamycin was injected intraperitoneally in doses (1, 1.5, 2, 3) mg/kg for three days, and (0.5) mg/kg for 10 days prior to STZ injection, where we picked the dose of 1 mg/kg body weight, injected 3 days prior to exposure to STZ for the R-STZ group. However, results revealed that inhibition of mTOR in the pancreas is not enough to guide the cells to be in an optimum autophagic state.

### 2.6. Experimental Induction of Diabetes Mellitus

Rats were made diabetic by a single I.P injection of 58 mg/kg body weight of STZ dissolved in citrate buffer (0.01 mol/L, pH 4.5); optimized by us after several trials [37]. Rats serving as controls were given the same volume of sodium citrate buffer. Animals were considered diabetic, when their fasting blood glucose (FBG) level exceeded 200 mg/dL after 72 h of exposure to STZ [38].

### 2.7. Urine Collection

At the end of the 6th week, animals from each group were kept individually in wire-bottom stainless-steel metabolic cages for collection of 24-h urine samples. 

During the period of urine collection, animals were fasted and allowed free access to water only. Urine samples were centrifuged at 600× g for 15 min at room temperature and the obtained supernatants were subjected to analysis for presence of albumin using diagnostic kit provided by Spectrum Diagnostics (Cairo, Egypt).

### 2.8. Blood Sampling

At the end of the experiment (7th week), all animals were anesthetized by diethyl ether, then the fasted blood samples were collected from retro-orbital plexus using capillary tubes. The blood allowed to clot in Eppendorf tubes, then centrifuged at 600× *g* for 15 min at 4 °C for serum separation for estimating insulin hormone and other routine biochemical tests.

### 2.9. Blood Chemistry

Kidney and liver function tests, uric acid, and other routine tests were measured in blood and serum enzymatically using kits obtained from Spectrum Diagnostics (Egypt) according to the reference method.

### 2.10. Tissue Sampling

Rats were dissected for isolating pancreas and the two kidneys. Each organ was washed immediately by ice-cold saline and then the pancreas was cut from its tail side [39] by a sharp razor blade into two portions. Every cut portion was standard among all rats in relation to the surface area and weight of pancreas in each individual rat.

The first portion was homogenized and determined tissue oxidative stress markers, malondialdehyde (MDA) and catalase (CAT), normalized for the total tissue protein.

The second portion was stored in a solution containing a cocktail of protease inhibitors for quantitative estimation of the autophagic marker LC3B-II in the pancreatic tissue as well as the apoptotic marker p53 expression level, and ABCB1 by WB analysis.

As for the kidneys, the adrenal glands were removed, then one kidney was stored in −80 and was used later for Western blot analysis. The other one was cut longitudinally to expose the great area of the organ that contains the majority of cells for exploring the histological features of the organ [40], then were preserved in 2% glutaraldehyde in 0.1 M phosphate buffer solution to be imaged for existence of autophagy, with TEM [41] or fixed in 10% buffered formalin and processed for histological study under light microscopy (hematoxylin and eosin stain, H&E) [42].

### 2.11. Western Blot (WB) Analysis

Pancreatic and renal proteins were extracted using radioimmunoprecipitation assay buffer, and protein concentrations were estimated by the Bradford method. 

20 μg of total protein from tissue homogenate were loaded into the wells of 10% (for detection of LC3B and p53) and 8% (for detection of ABCB1) SDS/PAGE gel, along with molecular weight marker. After 1 h of running the gel at 100 V, the migrated proteins on the gel were electrophoretically transferred to polyvinylidenene difluoride membranes that were activated with methanol for 1 min and rinsed with the transfer phosphate buffer saline before preparing the stack. Membranes were then incubated at room temperature for 2 h with 5% non-fat dry milk for 2 h at room temperature for blocking of non-specific binding sites, and then incubated overnight incubation at 4 °C with the primary antibodies; anti-LC3B (1:2000, Abcam, UK), anti-p53 (1:1000, Abcam, UK), and anti-ABCB1 (1:1000, Abcam, UK). After washing, the membranes were incubated with horseradish peroxidase-conjugated secondary antibody (1:5000) for 2 h at room temperature. The blots were developed using an enhanced chemiluminescent assay. Scanned images of the developed blots were automatically detected with image scanning and the optical density for each band was measured using Image Lab software (version 4.0, Bio-Rad, Hercules, CA, USA) after data were normalized to an internal control β-actin by Gel Doc™ EZ 3.0 [43]. 

### 2.12. Transmission Electron Microscope (TEM)

Thin slices of kidney were fixed in 2.5% cold glutaraldehyde then washed three times with phosphate buffer saline (pH 7.2) and post fixed in cold 1% osmium tetroxide for 2 h. Subsequently, the specimens were washed in buffer, dehydrated in a graded series of ethanol and acetone, and then embedded in epoxy resin. Ultrathin sections (50–80 nm) were cut with ultramicrotome, collected on copper grids and stained with 1% uranyl acetate and 0.2% lead citrate to be examined by TEM in the Regional Center for Mycology and Biotechnology, Al-Azhar University [44] Autophagosomes were observed by TEM (Wenzhou Medical University, H-7500, HITACHI, Tokyo, Japan) and imaged. Autophagosome structures were characterized by material surrounded by a double-layered membrane, with a higher electron density compared with the cytosol [45].

### 2.13. Statistical Analysis

Data are presented as means ±SD. The Chi-Square test was used for determining the incidence of DM in all group. Results of STZ-treated groups (R-STZ and S-STZ) were compared using Welch's *t*-test, after exclusion of non-diabetic rats. Statistical significance was acceptable to a level of *p* < 0.05. Data analysis was performed using GraphPad Prism 8.1.

## 3. Results

### 3.1. DM Markers

**Figure 1:**
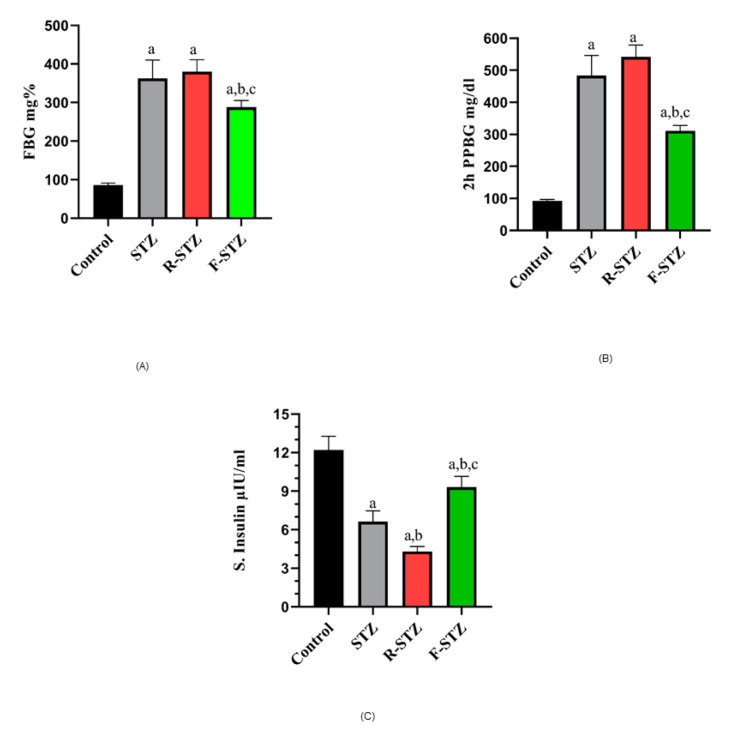
Blood glucose estimation in different experimental groups (**A**) FBG (mg/dl); (**B**) 2 h postprandial blood glucose (mg/dl); (**C**) Fasting serum insulin level (μIU/ml) in the different studied groups. (a) Significant difference from normal control group at *p* < 0.05; (b) Significant difference from STZ group at *p* < 0.05; (c) Significant difference from R-STZ group at *p* < 0.05.

The data compiled in Figure 1 showed that diabetic rats from the fasting group revealed a significant decrease in their FBG (A) and post prandial glucose level (B) from STZ control group. In addition, fasting serum insulin (μIU/ml) (C) was increased in rats subjected to fasting from STZ control group (*p* < 0.05), but still significantly less than the level of the normal group. In contrast to F-STZ, rapamycin-treated STZ group showed significant decrease in their fasting serum insulin level from both normal and STZ control groups. This was also the case for lipids profile data shown in (Table 1).

### 3.2. Kidney and Liver Function Tests

Rapamycin administration showed significant increase in serum urea compared to normal control group and other STZ groups. In addition, fasting model showed (*p* < 0.05) low serum creatinine level comparing to STZ control and rapamycin-treated STZ group that showed marked high level even from the STZ control group. The integrity of glomeruli of diabetic fasting rats, as compiled in Table 2, revealed no deterioration, as the level of urinary albumin was close to that of normal control group with no significant change. In contrast to diabetic fasting model, other STZ-treated rats showed a dramatically high amount of microalbuminuria. 

The excretory capacity of liver cells among the normal control group and diabetic rats of the fasting model were similar to each other with no significant change in total or direct serum bilirubin level. On the other hand, the rapamycin model exhibited the worst handling way of bilirubin by hepatocytes, as the serum total bilirubin level was remarkably the highest among all group of the experiment. Additionally, the serum direct bilirubin level in diabetic rats receiving rapamycin was significantly lower compared with the rest of the groups (Table 2). However, the synthetic capacity of the liver was approximately the same in all groups except rapamycin-treated STZ group, which showed significant low level of serum albumin from normal control group.

### 3.3. Nucleic Acid Integrity

The extent of STZ toxicity against nucleic acid was monitored in serum through measuring the level of serum uric acid, which demonstrated high significant serum uric acid levels in all STZ-treated groups compared to the normal control group. The level however, was the lowest in the fasting model in comparison to the STZ control and rapamycin groups (*p* < 0.05) (Table 2).

### 3.4. Tissue Oxidative Stress Estimation

**Figure 2:**
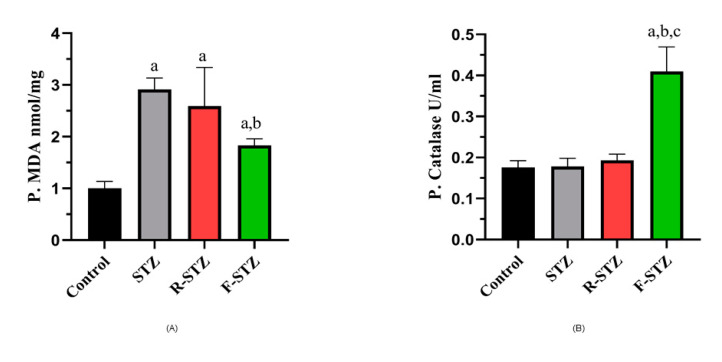
Pancreatic oxidative stress markers in all studied groups (**A**) Pancreatic MDA (mmol/mg); (**B**) Pancreatic CAT (U/ml). (a) Significant difference from normal control group at *p* < 0.05; (b) Significant difference from STZ group at *p* < 0.05; (c) Significant difference from R-STZ group at *p* < 0.05.

A dramatic increase in pancreatic MDA level in diabetic rats of the three STZ-treated groups was observed, with much more elevation (*p* < 0.05) in STZ control and rapamycin group over the level in fasting diabetic rats (Figure 2A). Restoration of the antioxidant capacity presented as CAT in the F-STZ in comparison to the control and STZ group (Figure 2B).

### 3.5. Cellular Autophagy

**Figure 3:**
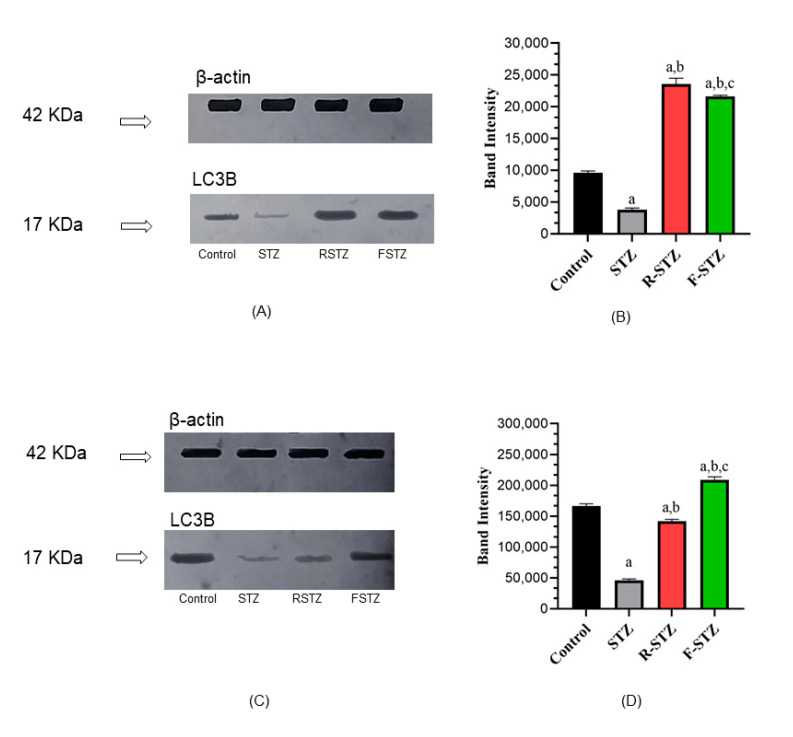
Cellular autophagy marker LC3B protein level in pancreatic and renal tissue samples of the study groups (**A**) Pancreatic representative WB; (**B**) Pancreatic densitometric quantification of WB, bars represent mean ± SD of band intensity (*n* = 5); (**C**) Renal representative WB; (**D**) Renal densitometric quantification of WB, bars represent mean ± SD of band intensity (*n* = 5). (a) Significant difference from normal control group at *p* < 0.05; (b) Significant difference from STZ group at *p* < 0.05; (c) Significant difference from R-STZ group at *p* < 0.05.

Protein expression of the autophagy marker LC3B, measured by WB, presented in Figure 3 was determined in pancreatic (Figure 3A,B) and renal tissues (Figure 3C,D) density and band intensity, that revealed a significant increase (*p* < 0.05) in rats’ tissues subjected to stress either by dietary restriction as fasting or mTOR inhibition via rapamycin administration to the R-STZ group, compared to rats of the normal control group. In contrast, rats subjected only to STZ showed a significant decrease of LC3B in comparison to the control group.

### 3.6. Cellular Apoptosis

**Figure 4:**
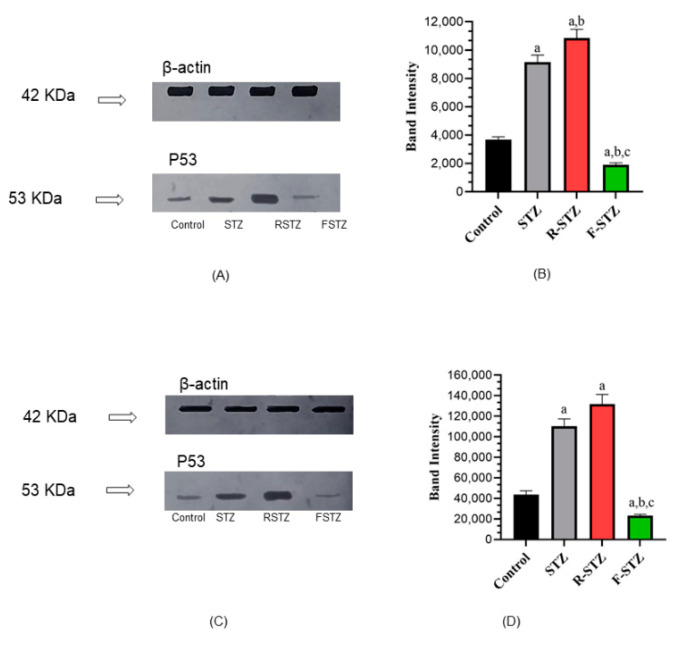
Cellular apoptosis marker p53 protein level in pancreatic and renal tissue samples of the study groups (**A**) Pancreatic representative WB; (**B**) Pancreatic densitometric quantification of WB, bars represent mean ±SD of band intensity (*n* = 5); (**C**) Renal representative WB; (**D**) Renal densitometric quantification of WB, bars represent mean ±SD of band intensity (*n* = 5). (a) Significant difference from normal control group at *p* < 0.05; (b) Significant difference from STZ group at *p* < 0.05; (c) Significant difference from R-STZ group at *p* < 0.05.

The apoptotic marker p53 expression level density and band intensity (Figure 4), measured by WB, in the pancreatic (Figure 4A,B) and kidney tissue (Figure 4C,D) samples of rats treated with rapamycin was the highest among the rest of study groups, with significant increase (*p* < 0.05) from the control and fasting groups. Unlike expected, the p53 level in rats subjected to fasting was significantly low, compared to the normal control group.

### 3.7. Cellular Permeability-Glycoprotein; ABCB1 Estimation

**Figure 5:**
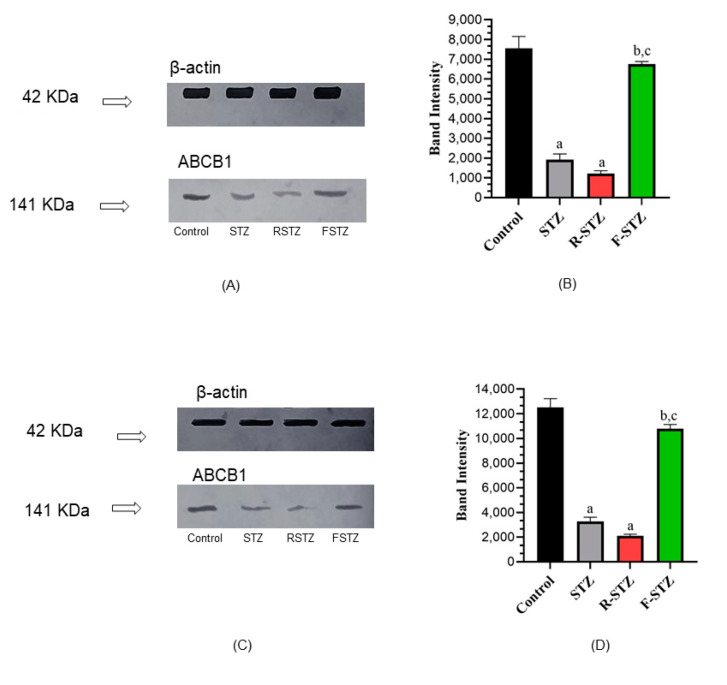
Cellular permeability-glycoprotein; ABCB1 level in pancreatic and renal tissue samples of the study groups (**A**) Pancreatic representative WB; (**B**) Pancreatic densitometric quantification of WB, bars represent mean ±SD of band intensity (*n* = 5); (**C**) Renal representative WB; (**D**) Renal densitometric quantification of WB, bars represent mean ±SD of band intensity (*n* = 5). (a) Significant difference from normal control group at *p* < 0.05; (b) Significant difference from STZ group at *p* < 0.05; (c) Significant difference from R-STZ group at *p* < 0.05.

In the current study (Figure 5), the three STZ treated groups showed low level of ABCB1 protein in either the pancreatic (Figure 5A,B) or the renal tissues (Figure 5C,D), with reference to the control group. However, the difference in the fasting group was non-significant compared with R-STZ treated group that exhibited a significant difference (*p* < 0.05) from the control group.

### 3.8. TEM Was Employed to Visualize the Occurrence of Autophagy as Evaluated by Autophagosome Formation

**Figure 6:**
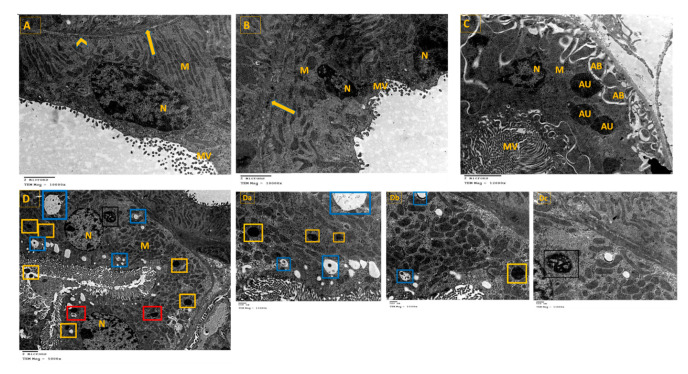
Effect of rapamycin and fasting on autophagosome formation in kidney tissue of rats after administration of STZ as viewed by TEM. (**A**) Renal proximal tubular cells of the control group showing normal nucleus (N), elongated abundant mitochondria (M) arranged perpendicular on trilamillar basal lamina (arrow), tight junctions (arrowhead). (**B**) Renal proximal tubular cells of STZ rats showing nucleus (N) with severe indentation of nuclear membrane, chromatin condensation, and margination suggestive of apoptotic processes. There is dilated intercellular space (arrow) showing swelling of endothelial cells (arrowhead). Mitochondria (M) are of different shapes and size which are also disoriented and showing swelling. (**C**) Renal proximal tubular cells of STZ rats that were administered rapamycin showing shrunken indentated nucleus (N), membrane blebbing, multiple apoptotic bodies (AB), and few autophagosomes (AU). There is great loss of cytoplasmic organelles, and the few abundant mitochondria (M) are disorganized. (**D**) Renal proximal tubular cells of fasting STZ rats showing normal nucleus (N), numerous intact elongated mitochondria (M), vacuoles (V), and tight intercellular space (arrow). Importantly, large number of cells had autophagosomes (yellow square) dispersed among them. Additionally, a higher magnification of parts of Figure 6 was provided as (**Da**–**Dc**) to demonstrate the four sequential steps in the autophagy process which are: (i) The initiation (formation of isolation membrane) (blue square), (ii) The formation of autophagosomes (yellow square), (iii) The fusion of autophagosome with lysosome (red square), (iv) The maturation of autophagosomes into autolysosomes (black square).

**Figure 7:**
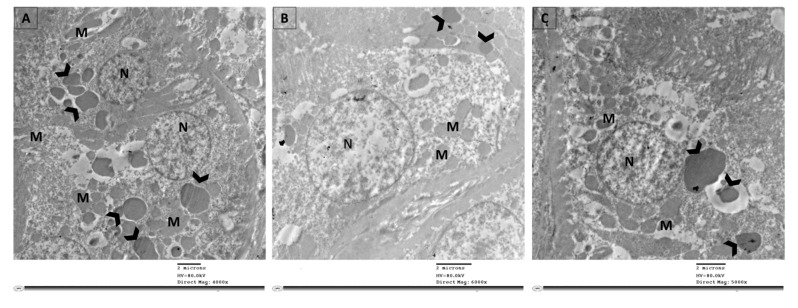
Selected micrographs by TEM of renal cells in the STZ group demonstrated active apoptosis with absence of autophagy. Active apoptosis appeared as apoptotic bodies (arrowhead) which are packages of fragmented intracellular components. Cytoplasmic gaps and swollen or irregular mitochondria (M) were abundant. (**A**) Apoptotic bodies (arrowhead) are mainly the results of splitting of the cellular content into distinct membrane-enclosed vesicles. (**B**) Apoptotic bodies as the result of plasma membrane blebbing (arrowhead). (**C**) Empty cytoplasm along with apoptotic bodies of larger size (arrowhead) and in close approximation to the nucleus (N) suggest inclusion of macromolecules such as proteins, lipids, RNA, and DNA.

**Figure 8:**
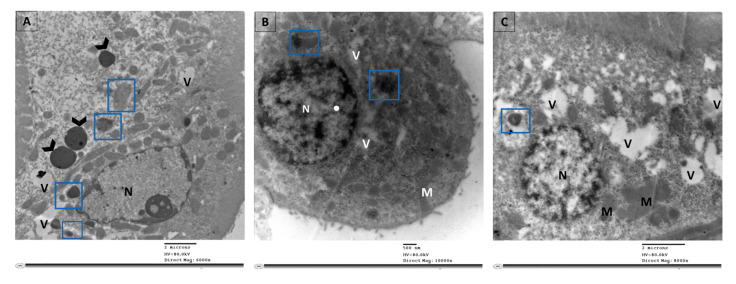
Selected micrographs by TEM of renal cells in the rapamycin groups displayed augmented apoptosis with little autophagy. Augmented apoptosis was evident as the chromatin condenses at the periphery of the nucleus (N) in addition to the abundance of apoptotic bodies (arrowhead) and vacuoles (V). The autophagic dynamic was disrupted as there were few phagocytic vesicles stuck as autophagosomes (blue square) with no autolysosomes. (**A**) A cellular behavior distinctive of apoptosis involves advanced margination and compacting of chromatin under the nuclear membrane, apoptotic bodies (arrowhead) and vacuoles (V). In addition, few autophagosomes (blue square) are found. (**B**) The collapse of cytoskeleton is indicated by diminished cell and shrunken nucleus (N), with an abundance of few number of vacuoles (V) and autophagosomes (blue square). (**C**) Atrophied nucleus with nuclear pores appears close to the areas of compact chromatin, extensive vacuolation (V) of the cytoplasm, fused mitochondria (M), and scarce autophagosomes (blue square).

**Figure 9:**
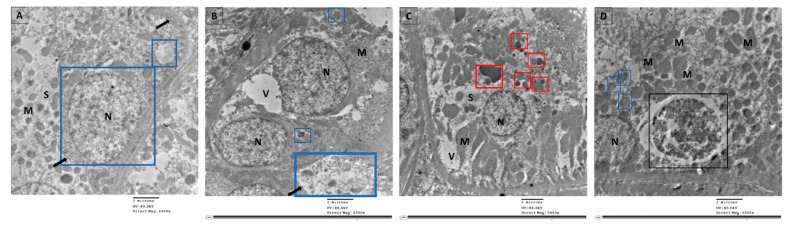
Selected micrographs by TEM of renal cells in the fasting group exhibited the four sequential steps in the autophagy process which are initiation and formation of isolation membrane (blue square) in (**A**), formation of autophagosomes (blue square) in (**B**), fusion of autophagosome with lysosome (red square) in (**C**), and maturation of autophagosomes into autolysosomes (black square) in (**D**). Signs of apoptosis were absent as there were no apoptotic bodies nor chromatin condensed at the periphery of the nucleus (N). In addition, newly formed mitochondria (M) were numerous, cytoplasmic space (S) were mostly in (**A**), and vacuoles (V) were observed in (**A**,**B**), while in (**C**) the cytoplasmic space (S) and the vacuoles (V) were less abundant.

Examination of ultrathin sections of renal cells from the control group revealed normal feature; normal nucleus (N), elongated abundant mitochondria (M) arranged perpendicular on trilamillar basal lamina (Figure 6A). On the other hand, renal proximal tubular cells of STZ group showed nucleus with severe indentation of nuclear membrane, chromatin condensation and margination suggestive of apoptotic processes (Figure 6B and Figure 7). Ultrathin sections of R-STZ group were characterized by numerous apoptotic bodies (AB) (Figure 6C and Figure 8). F-STZ, in contrast to the other STZ-treated group, revealed normal nucleus, normal intact mitochondria, vacuoles and lysosomes, and few autophagosomes (AU) (Figure 6D and Figure 9).

### 3.9. Histopathological Examination (H&E)

**Figure 10:**
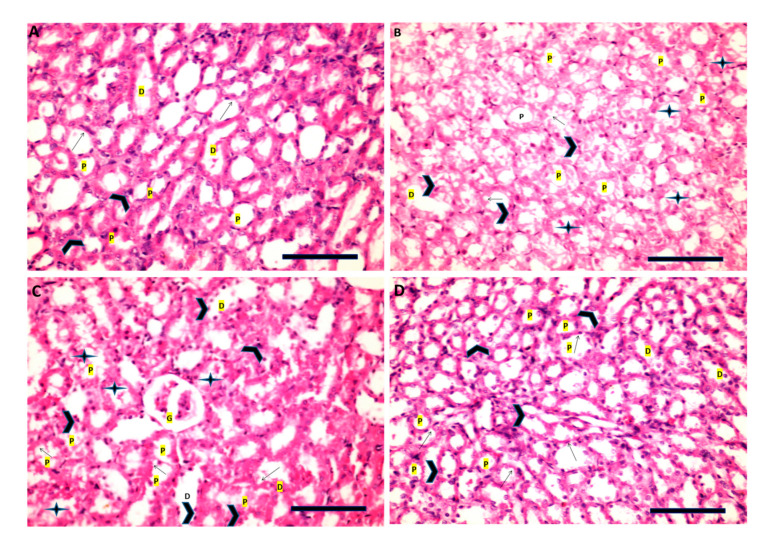
Renal tissues as stained by H&E (scale bar, 100 µm) for STZ rats after administration of rapamycin or after fasting. (**A**) Control rats are having normal architecture of proximal tubules (P) with intact brush border (arrow), and vesicular nuclei (arrowhead) of the epithelium lining, intact epithelium of the distal convoluted tubules (D) (H&E, ×100). (**B**) STZ rats having atrophied renal proximal (P) and distal (D) tubules with pyknotic nuclei (arrowhead) and loss of brush border (arrow) (H&E, ×40). (**C**) STZ rats that were administered rapamycin showing diffuse atypical glomerules (G), necrotic vacuolated epithelial cells of renal proximal (P) and distal (D) tubules with some hyaline cast in the lumen (star), pyknotic nuclei (arrowhead) and loss of brush border (arrow) (H&E, ×100). (**D**) Fasting STZ rats showing recovery of proximal tubules (P) with normal brush border (arrow), and vesicular nuclei (arrowhead) of the epithelium lining, intact epithelium of the distal convoluted tubules (D) (H&E, ×100).

Sections of renal cells from the control group showed normal architecture of proximal tubules (P), with intact brush border and vesicular nuclei (Figure 10A). On the other hand, examination of renal cells of the STZ rats, showed atrophied proximal and distal tubules (D), with pyknotic nuclei (Figure 10B). The renal cells of R-STZ group showed diffuse atypical glomerulus (G), necrotic vacuolated epithelial cells of proximal and distal tubules (Figure 10C). However, in the F-STZ group the proximal tubules recovered with normal brush border and vesicular nuclei (Figure 10D).

## 4. Discussion

Autophagy could be inhibited under the sequelae of diabetic settings [46], thus induction of autophagy appears as an attractive way to prevent and treat DM-related complication(s) as DN. 

In the current study, we pharmacologically induced autophagy via rapamycin administration; through the inactivation of mTOR pathway [47], and naturally through fasting [48], in an STZ rat model of DM.

STZ rat model to induce DM, resulted in deleterious effects on pancreatic metabolic parameters, and were further reflected into systemic metabolic and functional disorders. Pancreatic cells of STZ rats showed an active state of oxidative stress as indicated by high MDA levels and a simultaneous inactive autophagy as shown by low LC3B levels compared to the control group. Furthermore, an increased protein expression of p53 indicated the risk of programmed cell death.

Simultaneously, significant elevated glucose and decreased insulin levels and mitochondrial dysfunction, was previously reported [49]. Collectively, these findings showed decreased β-cells mass and function. Therefore, as a DM treatment option, maintenance of pancreatic β-cells could be achieved via enhancing β-cells defense power against destructive matters by inducing autophagy. Nevertheless, the subsequent risk of programmed cell death (apoptosis) might arise as occurred in the rapamycin-treated diabetic rats’ group. Accordingly, an optimal induction of autophagy is the key to gain the benefits of this vital biological process [50], with an indirect activation of CAT biosynthesis that is normally expressed in low levels in the pancreatic β-cells [51].

As STZ-treated-rats in our study, had renal and metabolic functions abnormalities, these were reflected as significantly higher levels of urea, uric acid, and creatinine with profound abundance of microalbuminuria. Low renal cell autophagic activity was aligned with an exacerbated oxidative stress, both of which are suspected to activate apoptosis and promote cell death [52]. This is in accordance to Kume et al., where diabetic conditions impaired autophagic activity, leading to aggravated renal injury in DN [53].

The permeability-glycoprotein; ABCB1 low protein levels in STZ-rats are in concert with a previous report, in which hyperglycemia inhibited Pgp expression [54], thus explaining the provoked STZ toxicity on both pancreatic and renal cells, due to inefficient foreign-substances pumping out of cells [24].

When rapamycin was administered to STZ-rats, a net negative effect on pancreatic and renal parameters occurred, where rapamycin induced autophagy concurrently with apoptosis [9,38]. This could be explained on the bases of that rapamycin inhibits cell proliferation and induces autophagy in both β-cells or renal cells [55]. The mechanism may be related to suppression of the mTOR signaling pathway. Rapamycin-mediated-mTOR-inhibition reverses the effect of permeability/trafficking glycoprotein(s), which is associated with increased autophagy, apoptosis, and reduced ABCB1 [56].

As the mTOR pathway controls the internalization and synthesis of different nutrient transporters and facilitates their subsequent absorption, mTOR has also been proposed as a regulator of cell membrane trafficking [57].

Intriguingly, active autophagy was not accompanied by active protein expression of ABCB1. Moreover, considering that rapamycin is a potential ABCB1 inhibitor, it is possible that autophagy plays a minor role in retrieving ABCB1, resulting in a minor effect on ABCB1 protein expression.

The outcome of this experiment could be considered a standard prophylaxis against DM-related complication(s), based on the individual activity level of the autophagic process. Therefore, impaired ABCB1 function is expected to increase toxic drug(s) effects or metabolite(s) accumulation in the kidney [57] and could, therefore, be involved in apoptosis implementation [58].

mTOR inhibitor drug rapamycin has previously been shown to stimulate β-cell autophagy [59]; however, reports on the effectiveness of this approach for DM treatment have been controversial. Few studies have reported that rapamycin is beneficial for glucose metabolism [60], while multiple reports have found that rapamycin reduces β-cells viability and impairs glucose tolerance [9,61].

In the current study, the pancreatic and renal cells of fasting STZ-rats had active autophagy. Energy deprivation was previously recognized to activate autophagy through SIRT1 which deacetylates multiple autophagy machinery components [62]. Additionally, ABCB1 showed an active expression in the pancreatic and renal cells of fasting STZ rats. Consistently, a study linked starvation with an increased expression of ABCB1 through both mitochondrial electron transport chain-derived and NADPH oxidase-4-induced oxidative signaling [63].

Interestingly, fasting-induced autophagy was associated with increased insulin secretion and mitigated hyperglycemia. This is contrary to the perception of fasting-induced autophagy as a checkpoint degradation pathway for wild-type proinsulin to prevent hypoglycemia under physiological conditions [47]. However, increased protein expression of ABCB1 in tandem with the ameliorated oxidative stress suggests that autophagy supports ABCB1 recovery and disables β-cells apoptosis, which eventually exerts a positive effect on insulin secretion, as well as a positive impact on oxidative stress/antioxidant contents. Thus, we identify fasting as a mechanism that regulates autophagy in favor of β-cell survival and insulin production. Recently, fasting has been proposed as a mechanism that manipulates autophagy to combat β-cell degeneration in DM [48].

As for renal functions, fasting STZ-rats displayed an improvement in the different examined kidney parameters. This finding suggests that active autophagy, along with enhanced protein expression of ABCB1, participated in a feedback loop to promote cellular homeostasis and survival.

In this sense, autophagy is proposed to enforce active expression of ABCB1 through recycling. On the other hand, ABCB1 complements the functions of fasting-induced autophagy, which is usually non-selective and simply phagocytoses in its surroundings to obtain energy. In this respect, ABCB1 is critical for resolving the stressful conditions and allowing perfect scenery for renal cells survival. The assumptions that ABCB1 is functional on the lysosomal membrane [64], and could act further as an adaptor protein for selective autophagic phagocytosis, need future studying.

Noteworthy, the protective power of fasting against DN injury is not restricted to its positive effect on autophagy, but also exhibits several beneficial effects including repression of NOX4 protein that is considered the main source of reactive oxygen species inside the kidneys [65]. In general, we can conclude that the major outcome of fasting is its ability to make cells adaptive and resistant to stress. It was previously shown that fasting supports the vitality and functionality of mitochondrial renal cells through providing good level of PGC1α; the key transcriptional regulator of mitochondrial biogenesis, and prevent renal damage and fibrosis [65]. In addition, the multisystemic beneficial effects of fasting affect the kidneys and the rest of organs in an indirect manner, as it reduces the gut microbiota [66] that interferes with the genetic background of the host, causing many human diseases [67].

## 5. Conclusions

Overall, therapies targeting autophagy are attractive options for treatment and prevention of diabetic renal complications. However, we found that certain approaches, namely rapamycin, had a net negative effect on pancreatic and renal parameters. While enabling autophagy, rapamycin induced apoptosis, so caution should be taken. On the other hand, fasting regulated autophagy in favor of β-cells survival and insulin production, while demonstrating improved renal parameters. In view of the fact that, unlike rapamycin, fasting enhanced the active expression of ABCB1 efflux protein, the potential ameliorative effects of fasting in DN require further elucidation.

## Figures and Tables

**Table 1:** Lipid profile of normal control and diabetic rats of STZ-treated groups (R-STZ and F-STZ).

Serum Parameters (mg/dl)\ Group (n)	Control (7)	STZ (6)	R-STZ (6)	F-STZ (6)
T.Cholesterol	119.6 ± 9.843	194.9 ± 4.889 ^a^	240.8 ± 50.09 ^a^	144.9 ± 4.02 ^a,b,c^
HDL-C	38.53 ± 3.327	18.06 ± 1.346 ^a^	16.46 ± 1.16 ^a^	26.73 ± 1.47 ^a,b,c^
LDL-C	52.57 ± 11.89	117.7 ± 5.38 ^a^	182.2 ± 51.68 ^a,b^	79.69 ± 4.52 ^a,b,c^
VLDL-C	28.54 ± 0.5757	59.06 ± 0.96 ^a^	42.07 ± 2.71 ^a,b^	38.46 ± 0.81 ^a,b,c^
TAG	142.7 ± 2.878	295.3 ± 4.82 ^a^	210.3 ± 13.54 ^a,b^	192.3 ± 4.06 ^a,b,c^

Values are expressed as mean ±SD, ^a^ Significant difference from normal control group at *p* < 0.05, ^b^ Significant difference from STZ group at *p* < 0.05, ^c^ Significant difference from R-STZ group at *p* < 0.05.

**Table 2:** Kidney and liver function tests of normal control and diabetic rats of STZ-treated groups (R-STZ and F-STZ).

Parameters\Group (n)	Control (7)	STZ (6)	R-STZ (6)	F-STZ (6)
S. Urea (mg/dl)	36.57 ± 1.25	117.9 ± 10.57 ^a^	147.3 ± 4.78 ^a,b^	65.05 ± 7.02 ^a,b,c^
S. Creatinine (mg/dl)	0.68 ± 0.08	1.947 ± 0.22 ^a^	2.3 ± 0.26 ^a,b^	1.008 ± 0.08 ^a,b,c^
Micro albuminuria (mg/24 h)	1.49 ± 0.04	2.493 ± 0.49 ^a^	2.962 ± 0.31 ^a^	1.580 ± 0.09 ^b,c^
S. Albumin (g/dl)	4.38 ± 0.36	4.160 ± 0.72	2.943 ± 0.299 ^a^	4.073 ± 0.279 ^c^
S. T.Bilirubin (mg/dl)	0.363 ± 0.042	0.787 ± 0.214 ^a^	1.132 ± 0.23 ^a,b^	0.365 ± 0.041 ^b,c^
S. D.Bilirubin (mg/dl)	0.064 ± 0.042	0.381 ± 0.033 ^a^	0.0225 ± 0.015 ^a,b^	0.104 ± 0.022 ^b,c^
S. Uric acid (mg/dl)	2.06 ± 0.12	5.492 ± 0.57 ^a^	5.09 ± 0.87 ^a^	3.05 ± 0.11 ^a,b,c^

Data are expressed as mean ±SD, ^a^ Significant difference from normal control group at *p* < 0.05, ^b^ Significant difference from STZ group at *p* < 0.05, ^c^ Significant difference from R-STZ group at *p* < 0.05.

## Data Availability

Not applicable.
